# In-line Filtration Decreases Systemic Inflammatory Response Syndrome, Renal and Hematologic Dysfunction in Pediatric Cardiac Intensive Care Patients

**DOI:** 10.1007/s00246-015-1157-x

**Published:** 2015-04-07

**Authors:** Michael Sasse, Friederike Dziuba, Thomas Jack, Harald Köditz, Torsten Kaussen, Harald Bertram, Philipp Beerbaum, Martin Boehne

**Affiliations:** Department of Pediatric Cardiology and Intensive Care Medicine, Hannover Medical School, Carl-Neuberg-Strasse 1, 30625 Hannover, Germany

**Keywords:** In-line filtration, Cardiac surgery, SIRS, Intensive care, Particle, Inflammation

## Abstract

Cardiac surgery with cardiopulmonary bypass (CPB) frequently leads to systemic inflammatory response syndrome (SIRS) with concomitant organ malfunction. Infused particles may exacerbate inflammatory syndromes since they activate the coagulation cascade and alter inflammatory response or microvascular perfusion. In a randomized, controlled, prospective trial, we have previously shown that particle-retentive in-line filtration prevented major complications in critically ill children. Now, we investigated the effect of in-line filtration on major complications in the subgroup of cardiac patients. Children admitted to tertiary pediatric intensive care unit were randomized to either control or filter group obtaining in-line filtration throughout complete infusion therapy. Risk differences and 95 % confidence intervals (CI) of several complications such as SIRS, sepsis, mortality, various organ failure and dysfunction were compared between both groups using the Wald method. 305 children (*n* = 150 control, *n* = 155 filter group) with cardiac diseases were finally analyzed. The majority was admitted after cardiac surgery with CPB. Risk of SIRS (−11.3 %; 95 % CI −21.8 to −0.5 %), renal (−10.0 %; 95 % CI −17.0 to −3.0 %) and hematologic (−8.1 %; 95 % CI −14.2 to −0.2 %) dysfunction were significantly decreased within the filter group. No risk differences were demonstrated for occurrence of sepsis, any other organ failure or dysfunctions between both groups. Infused particles might aggravate a systemic hypercoagulability and inflammation with subsequent organ malfunction in pediatric cardiac intensive care patients. Particle-retentive in-line filtration might be effective in preventing SIRS and maintaining renal and hematologic function. In-line filtration offers a novel therapeutic option to decrease morbidity in cardiac intensive care.

**Electronic supplementary material:**

The online version of this article (doi:10.1007/s00246-015-1157-x) contains supplementary material, which is available to authorized users.

## Introduction

Cardiac surgery with cardiopulmonary bypass (CPB) is associated with the development of systemic inflammatory response syndrome (SIRS) [6, 10]. Additionally to the inflammatory response due to surgical trauma, several inflammatory processes are triggered by the CPB. Hypothermia, contact of blood with foreign surfaces of the CPB circuit or ischemia–reperfusion injury due to aortic cross-clamping and endotoxins generated by splanchnic hypoperfusion all stimulate multiple inflammatory cascades with humoral and cellular reactions [10]. SIRS often leads to organ dysfunction or failure [9, 31], increased mortality and prolonged stay on intensive care unit (ICU) [27, 31]. Numerous strategies have been developed to minimize the inflammatory response to CPB such as corticosteroids, leukocyte depletion, hemofiltration, miniaturized CPB circuits, off-pump surgery and alterations of the coagulation/anticoagulation management [6, 10]. So far, all current strategies focused on modification of pre- and intraoperative factors. However, the inflammatory processes may be maintained or even intensified by further treatment on ICU. So far, no preventive strategy has been established for ICU management.

Particles arising from infusion therapy on ICU may aggravate the inflammatory response. Particles have been shown to generate thrombosis [34], impair microcirculation [29] and modulate immune response [19]. Sources of particles include components of infusion systems, incomplete reconstitution of solutions or drug incompatibility reactions [19, 30]. Up to one million particles may be infused per patient per day [19, 20]. In-line filters incorporated into infusion lines retain particles and thereby nearly entirely prevent their infusion [19, 29]. In a prospective, randomized, controlled trial (ClinicalTrials.gov number; NCT00209768) including 807 critically ill children, we have previously demonstrated that in-line filtration reduced the composite endpoint of severe complications including sepsis, SIRS and organ failure [7, 20]. Additionally, SIRS and several organ dysfunctions were reduced as single incidence [7, 20]. Moreover, length of stay (LOS) on pediatric intensive care unit (PICU) and duration of mechanical ventilation (MV) were also significantly shortened [20].

In the previous study population, we now analyzed the effect of in-line filtration on major complications in the subgroup of cardiac patients (*n* = 305), which has not been described in detail before. Risk differences of several complications such as SIRS, sepsis, mortality, various organ failure and dysfunction were compared between both groups. Furthermore, LOS on PICU and time of MV were analyzed.

## Materials and Methods

### Study Design

This was an ancillary study to a single-center, prospective, randomized, controlled trial regarding in-line filtration which was conducted between February 2005 and September 2008 on an interdisciplinary PICU of a German university hospital (ClinicalTrials.gov number; NCT00209768). The current investigation focused on the subgroup of children with cardiac disease, which have not been investigated in detail before. Due to safety reasons, use of in-line filters could not be blinded. The local Ethical committee approved the research protocol. Upon admission, written informed consent was obtained for each child from its legal guardians.

### Patient Enrollment and Randomization

For the complete study group, patient enrollment and randomization have been described previously [7, 20]. Patients below 18 years of age admitted to PICU were assessed for eligibility. Exclusion criteria comprised expected death within 48 h of admission, recruitment for other trials, absence of intravenous infusion therapy and absent parental informed consent. According to a computer-generated simple unrestricted randomization list, patients were allocated to either control or filter group. Discharge from PICU within 6 h and discontinuation of intervention were predefined as exclusion criteria. For the complete study group, 807 patients (*n* = 406 control, *n* = 401 filter group) were included in the final analysis [7, 20]. Of these patients, 305 children (*n* = 150 control, *n* = 155 filter group) suffered from cardiac diseases.

### In-line Filtration

First, a standardized infusion therapy was implemented for all patients before starting the study [7, 20]. Drug incompatibilities were avoided using a computer-based program (KiK 3.0; oData, Rastede, Germany) [30]. Solutions and medications were produced according to manufacturer’s guidelines. In order to guarantee chemical stability and aseptic standards, a centralized intravenous additive service provided parenteral nutrition and specific drugs (special antibiotics, antiviral drugs, antimycotics and chemotherapy). Administration sets for lipid-containing admixtures were replaced every 24 h, others every 72 h as recommended by the Robert Koch Institute [23]. To assure an appropriate and careful handling of in-line filters, all nurses and physicians received an intensive training.

Visible in-line filters were obligate for safe drug application and surveillance of imminent blockage. Within the filter group, in-line filters were placed in each lumen of central and peripheral venous catheters throughout complete infusion therapy [7, 20]. All medications and solutions apart from blood, plasma proteins or fresh frozen plasma were applied via an in-line filter. Aqueous solutions were administered via 0.2-µm-pore-size positively charged filters (ELD96LLCE/NOE96E; Pall, Dreieich, Germany) and lipid-containing admixtures via 1.2-µm-pore-size filters (Intrapur Lipid/Intrapur Neonat Lipid; B Braun, Melsungen, Germany). In-line filters were routinely exchanged after 24 (Intrapur Lipid/Intrapur Neonat Lipid) or 72 h (ELD96LLCE/NOE96E), or after blockage.

### Data Collection

On admission, demographic and clinical data including Risk Adjustment in Congenital Heart Surgery 1 (RACHS-1) [22] were recorded in a database. Patients with combined surgical procedures were assigned to the risk category of the most complex procedure [22]. Clinical data were documented at least every hour. Laboratory tests were routinely conducted on admission and when clinically indicated. In case criteria for endpoints were fulfilled, this was recorded in the database, which was thoroughly reviewed for consistency. Any complication prior to PICU stay or present on admission was not considered. Finally, investigators blinded for randomization checked database entries.

### Endpoints

Endpoints for the complete study group have been published elsewhere [7, 20]. In this investigation, occurrence of the following major events was analyzed in the subgroup with cardiac diseases: SIRS, sepsis, mortality, organ failure (circulation, lung, liver and kidney) or organ dysfunction (cardiovascular, respiratory, neurologic, hematologic, renal and hepatic). All complications were defined according to accepted pediatric consensus recommendations [3, 5, 16, 17, 32]. SIRS, sepsis and organ dysfunction (Electronic Supplementary Material) were defined according to the International pediatric sepsis consensus conference (IPSCC) 2005 [16, 17] and acute respiratory distress syndrome (ARDS) analogous to the American-European Consensus Conference [5]. The Pediatric Acute Liver Failure Study group determined criteria for acute liver failure [32]. Circulatory failure was considered as necessity for any vasoactive or inotropic drug (epinephrine, norepinephrine, dobutamine and dopamine) to maintain blood pressure above the fifth age-specific percentile [16]. Definition of acute renal failure was based on the Pediatric-modified Risk, Injury, Failure, Loss, End-stage kidney disease (pRIFLE) criteria [3]. Finally, mortality rates, LOS on PICU and duration of MV were assessed for both groups [7, 20].

### Statistical Analysis

Statistical analysis has been described previously [20]. Briefly, due to low mortality rates on PICU, a decrease in the overall complication rate of major events such as SIRS, sepsis, thrombosis and several organ failures was defined as primary endpoint for the complete study group. Therefore, the initial study was designed on an intention-to-treat basis to determine a reduction from 40 to 30 % in the complication rate of major events for the filter group as the primary endpoint [20]. Size and power of the trial were not calculated for recognition of a single reduction in a complication for a subgroup.

In this subgroup analysis, demographic data were compared between control and filter groups using *t* test for equality of means. All tests were two-sided. *P* < 0.05 was considered to indicate statistical significance. Data for duration of MV and LOS on PICU are stated as median and range, all other data as mean ± SD (95 % CI). The log-rank test was applied to compare duration of MV and LOS on PICU. For each endpoint, the risk and its differences between the both groups including the 95 % confidence interval (CI) were calculated by the Wald method. If the 95 % CI laid on either side below zero, a statistically significant difference between both groups was considered. To exclude any potential influence of differences in baseline characteristics between both groups, we performed a multivariable analysis with an adjustment for demographic parameters. For the multivariable analysis, we used generalized linear models for estimating risk differences of the association between filter group and selected outcomes. We did not find any influence on outcome parameters of potential differences in baseline characteristics. The results for the adjusted risk differences are presented in Table 2, and the data for the crude risk differences are additionally presented in the Electronic Supplementary Material. Statistical analysis was performed using Predictive Analysis Software for Windows (SPSS/PASW), version 18.

## Results

### Subjects

305 children, assigned to either control (*n* = 150) or filter group (*n* = 155), were included in the final analysis (Table 1). Almost four out of five were admitted after cardiac surgery (*n* = 118 control, *n* = 120 filter group). Most of them received surgery of congenital heart disease (CHD) with cardiopulmonary bypass (*n* = 102 control, *n* = 101 filter group) (Table 1). There were no significant differences between both groups regarding baseline demographic characteristics, RACHS-1, lowest temperature during CPB, aortic cross-clamping or cardiopulmonary bypass times (Table 1).

**Table 1:** Baseline characteristics of patients

Characteristics	Control group (*n* = 150)	Filter group (*n* = 155)	*P* value
Age (years)	3.1 ± 4.6	3.7 ± 5.5	0.29
Weight (kg)	13.1 ± 14.9	15.5 ± 18.8	0.21
Sex (no.)			
Male	92	100	0.64
Female	58	55	
Cardiac surgery-no.			
No	32	35	
Yes	118	120	0.58
CHD with CPB	102	101	
*Cardiac surgery*			
Preoperative cyanotic CHD (postoperative persistent)			
Yes	48 (10)	45 (11)	
No	70 (108)	75 (109)	0.69 (1.00)
Total-no.	118	120	
RACHS-1			
Risk category (no.)			
1	19	16	
2	45	53	
3	43	36	
4	4	10	
5	0	0	
6	4	4	0.40
Total-no.	115	119	
CPB time (min)	118.4 ± 62.2	113.9 ± 52.3	0.54
Nadir blood temperature (C°) during CPB	23.6 ± 5.4	24.0 ± 4.8	0.60
Aortic XCT (min)	47.9 ± 27.9	44.8 ± 26.7	0.43
Total-no.	95	95	
*Noncardiac surgery*			
Arrhythmia/conductive disorder	5	6	
Cardiomyopathy/myocarditis	6	4	
Cyanotic CHD	7	9	
Acyanotic CHD			
Left-to-right shunt lesion	1	3	
Obstructive lesion	6	6	
Pulmonary arterial hypertension	4	4	
Others	3	3	
Total-no.	32	35	0.95

Table shows allocation of patients to control and filter groups according to demographic characteristics, cardiac surgery and noncardiac surgery entities. None of the differences between the two groups were significant. Data are reported as mean ± SD, or as number (no.) of patients when indicated

*RACHS*-*1* Risk Adjustment in Congenital Heart Surgery 1, *CHD* congenital heart disease, *CPB* cardiopulmonary bypass, *Aortic XCT* aortic cross-clamping time

### SIRS, Sepsis, Mortality and Organ Failure

**Fig. 1:**
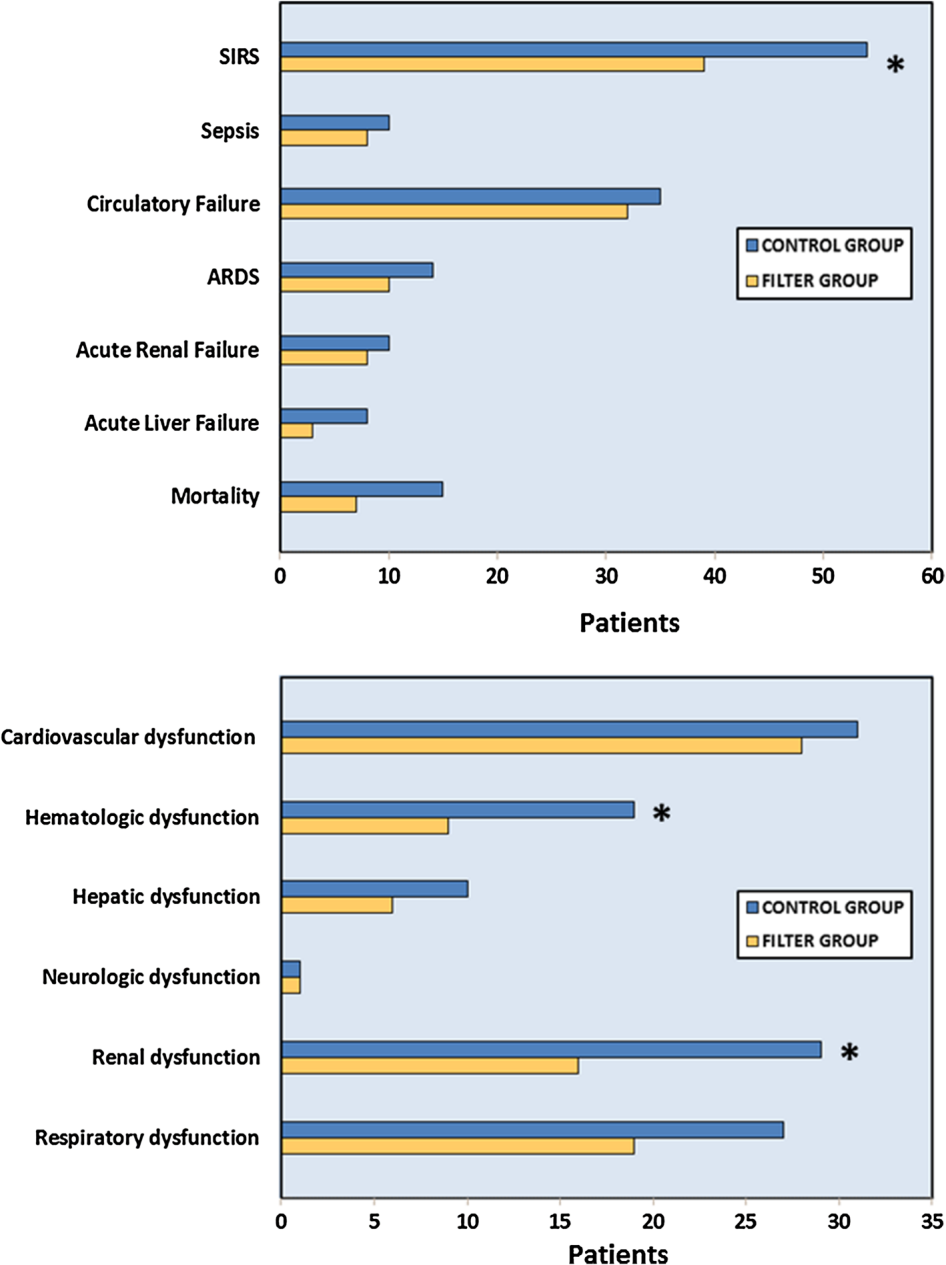
Incidence of endpoints in control (*blue columns*) and filter group (*yellow columns*). Figure presents incidence of endpoints between control (*blue columns*) and filter group (*yellow columns*). *Upper panel* shows incidence of systemic inflammatory response syndrome (SIRS), sepsis, organ failure (circulation, lung, kidney and liver) and mortality. *Lower panel* displays occurrence of different organ dysfunctions (cardiovascular, hematologic, hepatic, neurologic, renal and respiratory). SIRS, renal and hematologic dysfunction were significantly reduced in the filter group (*asterisk*). If the 95 % CI did not embrace zero, a statistically significant difference between both groups was considered

The risk difference of SIRS between the control [36.0 % (*n* = 54)] and filter group [25.2 % (*n* = 39)] was significantly different [risk difference = −11.3 % (95 % CI −21.8 to −0.5 %), (Table 2; Fig. 1, upper panel)]. Though the incidence of any other complication was decreased in the filter group, no statistically significant risk differences could be demonstrated for sepsis, mortality, circulatory failure, ARDS, acute liver and acute renal failure (Table 2; Fig. 1, upper panel).

**Table 2:** Endpoints

Endpoints	Control group (*n* = 150)	Filter group (*n* = 155)	Risk difference (%)	95 % CI
*SIRS, sepsis, organ failure and mortality*				
SIRS	54	39	−11.3	−21.8 to (−0.5)*
Sepsis	10	8	−2.0	−6.5 to 2.5
Circulatory failure	35	32	−2.5	−11.8 to 6.7
ARDS	14	7	−3.5	−9.0 to 1.9
Acute renal failure	10	8	−1.6	−7.0 to 3.9
Acute liver failure	6	2	−2.3	−5.3 to 0.6
Mortality	15	7	−5.8	−11.5 to 0.1
*Organ dysfunction*				
Cardiovascular dysfunction	31	28	−2.3	−11.0 to 6.3
Hematologic dysfunction	19	9	−8.1	−14.2 to (−0.2)*
Hepatic dysfunction	10	6	−2.8	−7.8 to 2.2
Neurologic dysfunction	1	1	0.0	−2.0 to 2.0
Renal dysfunction	29	16	−10.0	−17.0 to (−3.0)*
Respiratory dysfunction	27	19	−5.6	−13.6 to 2.4

Table shows incidence of different complications in control and filter groups, risk differences and corresponding 95 % confidence interval (CI) according to Wald method

Calculation of a *P* value was statistically inappropriate in a subgroup analysis. Therefore, risk differences in incidence rates and their corresponding 95 % CI were determined. A 95 % CI on either side below zero indicated a statistically significant difference between both groups (*). SIRS, renal and hematologic dysfunction were significantly reduced in the filter group

### Organ Dysfunction

Patients receiving in-line filtration developed significantly less renal dysfunction [10.3 % (*n* = 16) vs. 19.3 % (*n* = 29); risk difference = −10.0 %; 95 % CI −17.0 to −3.0 %; filter vs. control group] (Table 2; Fig. 1, lower panel) and hematologic dysfunction [5.8 % (*n* = 9) vs. 12.7 % (*n* = 19); risk difference = −8.1 %; 95 % CI −14.2 to −0.2 %; filter vs. control group]. No relevant risk differences were demonstrated for respiratory, cardiovascular, hepatic and neurologic dysfunction between both groups (Table 2; Fig. 1, lower panel).

### Duration of MV and LOS on PICU

Though not statistically significant, LOS on PICU was reduced by almost 1 day in the filter group [5.86 days (95 % CI 4.91 − 6.82) vs. 4.95 days (3.63 − 6.27); control vs. filter group; *P* = 0.109], (Table 3). Duration of MV was approximately 15 h shorter within the filter group [33.0 h (95 % CI 12.7 − 53.3 h) vs. 18.3 h (4.6 − 31.9 h)], but this difference did not reach statistical significance as well.

**Table 3:** Duration of mechanical ventilation (MV) and length of stay (LOS) on PICU

Endpoints	Control group (*n* = 150)	Filter group (*n* = 155)	*P* value
LOS on PICU (days)	5.86 (4.91–6.82)	4.95 (3.63–6.27)	0.109
Duration of MV (h)	33.0 (12.7–53.3)	18.3 (4.6–31.9)	0.076

Table shows length of stay (LOS) on PICU and duration of mechanical ventilation (MV) for control and filter groups

## Discussion

Recently, we conducted a prospective, randomized, controlled trial to evaluate the effect of in-line filtration on major complications in critically ill children [7, 20]. The current investigation focused on the subgroup of children with cardiac diseases in the previously published study population [7, 20]. This subgroup of cardiac patients has not been investigated in detail before. In addition to our previous data [7, 20], we further characterized underlying cardiac diagnoses, the procedural risk for cardiac surgery and intraoperative data. Until now, no study has ever investigated the effect of in-line filtration retaining particles arising from infusion solutions during stay on cardiac PICU.

Since use of a contingency table or calculation of a *P* value is statistically inappropriate in a subgroup analysis, we computed risk differences and its 95 % confidence interval (CI) for each endpoint. A statistically significant difference between control and filter group was assumed, if the 95 % CI of the risk differences did not contain zero. The present analysis thereby comprises descriptive statistics.

In the light of this methodological approach, our main findings suggest that in-line filtration is highly effective in preventing the occurrence of SIRS in cardiac intensive care patients. Moreover, in-line filtration seemed to preserve hematologic and renal organ function. Length of stay on ICU and the duration of MV as well as all other complications were observed to be decreased in the filter group, albeit not reaching the level of significance as defined above.

For the evaluation of SIRS, we applied the generally accepted pediatric consensus criteria [17]. So far, no other trial has assessed the incidence of SIRS in critically ill children with cardiac diseases. Our study revealed that SIRS was a major problem on a cardiac PICU. According to our data, more than one-third of all patients experienced at least one episode of SIRS. In-line filtration effectively reduced the incidence of SIRS and thereby might lower the morbidity of cardiac intensive care patients. Patients suffering from SIRS have a higher risk to develop sepsis [28], ARDS, disseminated intravascular coagulation, acute renal failure or even die of SIRS [27]. Moreover, according to our data [20] and also demonstrated by others, SIRS is associated with both prolonged stay on PICU and extended MV [31]. In-line filtration represents the first preventive strategy reducing a systemic inflammation and comorbidities on PICU.

The control group suffered from a higher incidence of hematological dysfunction characterized by thrombocytopenia and coagulopathy. As patients with CHD per se and even more pronounced in cyanotic CHD show an altered hemostatic physiology with decreased concentrations of coagulation factors and abnormalities of platelets [14], an additional infusion of particles obstructing small capillaries may worsen the preexisting coagulopathy [34, 35]. No cardiac right-to-left shunt provided, in patients without particle-retentive in-line filters, the lung capillaries are the first endogenous filters for intravenously infused particles [15, 34, 35]. Particles trapped in lung capillaries, form occlusive microthrombi and induce an activation of complement, platelets and neutrophils [34, 35]. But, blockage of vessels is not only restricted to the lung. Even without a relevant cardiac right-to-left shunt, intravenously injected particles are distributed from the lung to the systemic circulation and several organs such as blood, liver, kidney and spleen [12, 29]. Here, they may induce a systemic hypercoagulability. These systemic effects on different organ systems might be even more pronounced in patients with CHD and right-to-left shunts. As the coagulation and inflammatory system are closely linked in multiple ways [25], this cross-link may contribute to a further aggravation of both hypercoagulation and SIRS. In our study, this deleterious linkage becomes clinically apparent in the control group: Patients without in-line filters suffered from a higher incidence of SIRS and coagulopathy. Especially in patients after cardiac surgery with CPB, the physiological hemostasis of the coagulation system is altered for several days postoperatively [8, 21] as the contact of blood to the artificial surfaces of the CPB circuit causes an activation and consumption of platelets and coagulation factors [8]. A further infusion of particles on ICU as in the control group may additionally trigger the coagulation cascade and contribute to the coagulopathy. This systemic hypercoagulability also plays a relevant role in the pathogenesis of microvascular impairment and organ failure at multiple sites [25].

As demonstrated in our study, cardiac patients who are not protected by particle-retentive in-line filters had a higher incidence of renal dysfunction. An infusion of particles may disturb the renal vascular integrity and enhance the susceptibility to organ dysfunction. Especially patients with cyanotic CHD have an increased risk of developing renal impairment [2]. Many studies have shown proteinuria, reduced glomerular filtration rate, and proliferation of renal tubular and glomerular cells in patients with CHD [2]. Contributing factors such as CPB may further augment the effects of particle infusion. Renal injury following CPB is frequent, and its pathogenesis is multifactorial [1]. Ischemia–reperfusion injury, oxidative stress, microembolization and the inflammatory response have been accused for the development of renal injury [1, 18]. The increased incidence of renal impairment may also contribute to a prolonged stay on PICU and extended MV as shown in children after cardiac surgery [26].

In the control group lacking in-line filters, particles obstructing lung capillaries trigger the complement system and activate platelets and neutrophils [15, 34, 35]. This inflammatory process may lead to an intensified and prolonged MV as shown for the control group in the main study population [20]. In patients after cardiac surgery with CPB, the effect of particle infusion may be even more pronounced and the pulmonary inflammation exaggerated. During CPB, the lungs are excluded from the circulation and remain ischemic and hypoxic for a long period [4]. The following reperfusion induces an inflammatory response with formation of free oxygen radicals and endothelial injury [4]. Beyond this local pulmonary inflammation, several inflammatory mediators released during CPB stimulate the proliferation of neutrophils and their migration to lung capillaries [4]. Here they additionally induce an endothelial cell swelling and an inflammation in the interstitial tissue and alveoli [4]. A further particle infusion may aggravate this postperfusion lung syndrome.

The differences in the clinical findings between the control and the filter group might be attributed to a further deterioration of microvascular perfusion by particulate infusion in the control group. Microcirculatory disturbance occurs frequently in critically ill patients and leads to organ malfunction [13]. With already preexisting microvascular impairment, an additional infusion of particles causes a further loss of microvascular density as demonstrated in preclinical animal studies [24, 29]. In the same experimental settings, use of particle-retentive in-line filters was able to completely prevent these effects [29]. Therefore, in-line filtration may account for the maintenance of microvascular integrity and thereby preserve the multiple organ functions within the interventional group. One could assume that any critically ill patient regardless the underlying disease would benefit from the potential protective properties of in-line filtration on microcirculation. Especially during cardiac surgery, both the inflammatory response to CPB and the hemodynamic and metabolic changes alter the microcirculatory perfusion and decrease capillary density [11, 13]: a condition that persists at least for the first 24 postoperative hours on ICU [11]. In this state, additional microcirculatory impairment may exceed the level of organ retrieval and organ dysfunction becomes clinically apparent. An early recovery of microcirculation is therefore associated with a faster restoration of organ function [33] in the interventional group.

## Limitations

Size and power of this investigation were computed for neither a reduction of a single complication nor a subgroup analysis. Since many complications were reduced in the in-line filter group yet without statistical significance, future studies comprising an appropriate sample size are required to confirm this trend. Particularly in patients with right-to-left shunts, a higher amount of infused particles enters directly the systemic circulation bypassing the lung as endogenous filter. Therefore, one would expect a higher incidence of malfunction of those organs supplied by the systemic circulation. Unfortunately, in our study, the number of patients with cyanotic CHD was too small to allow any reliable statistical evaluation. Further prospective studies will be needed to corroborate this hypothesis.

Placement of in-line filters into infusion lines was not blinded. This may have affected handling with drugs and infusion solutions. A study design with unmasked, visible in-line filters was obligate for a safe drug application and monitoring of upcoming clogging of the filter membrane.

## Conclusion

Particulate contaminations of infusion solutions pose an additional risk to cardiac intensive care patients. Prevention of particulate infusion by in-line filtration reduces the incidence of SIRS and preserves renal and hematologic function. Further prospective randomized trials are needed to confirm our results.

## Electronic supplementary material

Supplementary material 1 (DOCX 277 kb)

## Abbreviations

ARDS: Acute respiratory distress syndrome
CHD: Congenital heart disease
CI: Confidence interval
CPB: Cardiopulmonary bypass
ICU: Intensive care unit
INR: International normalized ratio
IPSCC: International pediatric sepsis consensus conference
LOS: Length of stay
MV: Mechanical ventilation
PICU: Pediatric intensive care unit
PIM: Pediatric index of mortality
RACHS-1: Risk Adjustment in Congenital Heart Surgery 1
RIFLE: Risk, Injury, Failure, Loss, End-stage kidney disease
SIRS: Systemic inflammatory response syndrome
