# Supplementary material for: In-line Filtration Decreases Systemic Inflammatory Response Syndrome, Renal and Hematologic Dysfunction in Pediatric Cardiac Intensive Care Patients
Source: Pediatr Cardiol. 2015 Apr 7;36(6):1270–8. doi: 10.1007/s00246-015-1157-x (PMC4495711; doi:10.1007/s00246-015-1157-x)
Supplement: Supplementary file 1 — Supplementary material 1 (DOCX 277 kb) [file 246_2015_1157_MOESM1_ESM.docx]

**Electronic Supplementary Material**

| **Cardiovascular** | Despite intravenous application of ≥40 ml/kg isotonic volume in 60 minutes persisting:   - Hypotension with BP <5^th^ percentile for age or systolic BP < 2 SD below normal for age   OR   - Vasoactive drug therapy to keep BP in normal range (dopamine >5µg/kg/min or epinephrine, norepinephrine, or dobutamine at any dose)   OR   - Two of the following   - Arterial lactate >2 times upper limit of normal  - Prolonged capillary refill >5 sec.  - Oliguria: urine output <0.5 ml/kg/h  - Metabolic acidosis (base deficit >5 mmol/l)  - Core to peripheral body temperature difference >3°C |
| --- | --- |
|  |  |
|  |  |
| **Hematologic** | - Thrombocyte count < 80.000/mm³ or decline of 50% in thrombocyte count from highest value recorded over the past 3 days (for chronic hematology/oncology patients)   OR   - International Normalized Ratio >2 |
| **Hepatic** | - Total bilirubin ≥ 4mg/dL (not applicable for newborn)   OR   - ALT 2 times upper limit of normal age |
| **Neurologic** | - Glasgow Coma Scale (GCS) ≤11   OR   - Acute change in mental status with decrease in GCS ≥3 points from abnormal baseline |
| **Renal** | Serum creatinine ≥ 2 times upper limit of normal for age or 2-fold rise in baseline creatinine |
|  |  |
| **Respiratory** | - Oxygenation index <300 in absence of cyanotic heart disease or preexisting lung disease   OR   - PaCO_2_ >65 mmHg or increase of >20 mmHg over baseline   OR   - Proven need or FiO_2_ >0.5 in order to maintain saturation ≥92%   OR   - Need for nonelective mechanical ventilation (invasive or non-invasive) |

Table 1: Criteria for organ dysfunction

*Table displays diagnostic criteria for cardiovascular, hematologic, hepatic, neurologic, renal and respiratory dysfunction according to the International pediatric sepsis consensus conference [11,14,18].*

*BP, blood pressure; GCS, Glasgow Coma Scale; ALT, alanine aminotransferase.*


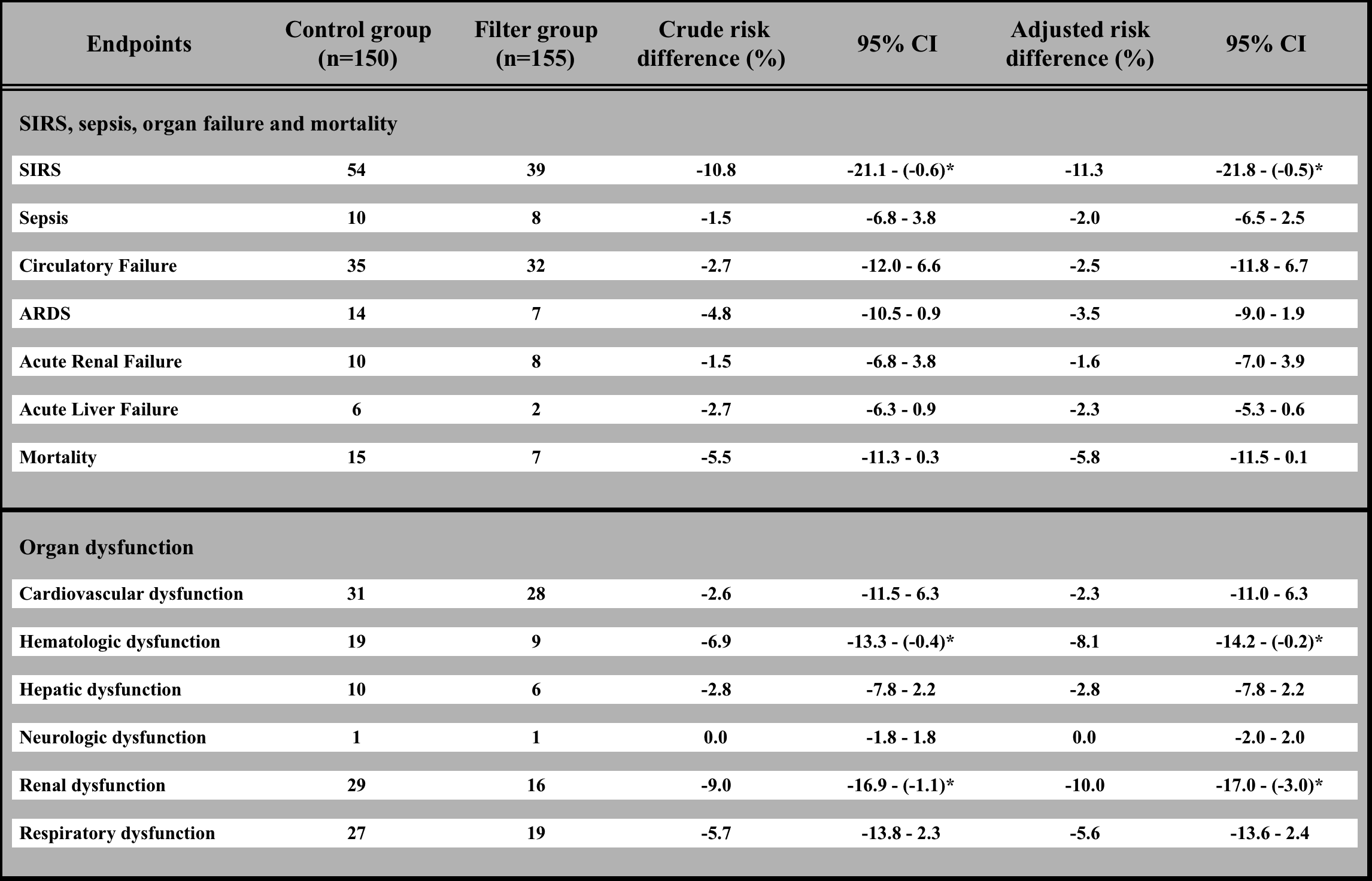


**Table 2: Endpoints**

*Table shows incidence of different complications in control and filter group, risk differences and corresponding 95% confidence interval (CI) according to Wald method. The adjusted risk differences are presented in the main manuscript. For further information and comparison the crude risk differences are additionally shown in this table.*

*Calculation of a P value was statistically inappropriate in a subgroup analysis. Therefore risk differences and their corresponding 95% CI were determined. A 95% CI on either side below zero indicated a statistically significant difference between both groups (*). SIRS, renal and hematologic dysfunction were significantly reduced in the filter group.*
